# 820. Clinical Outcomes in Patients with Candidemia with and without Infectious Diseases Consult

**DOI:** 10.1093/ofid/ofad500.865

**Published:** 2023-11-27

**Authors:** Michelle Grace Guerrero, Ty Drake, Clare N Gentry, Jessica T Babic

**Affiliations:** Memorial Hermann - Texas Medical Center, Houston, Texas; Memorial Hermann - Texas Medical Center, Houston, Texas; UTHealth Houston McGovern Medical School, Houston, Texas; Memorial Hermann-Texas Medical Center, Houston, Texas

## Abstract

**Background:**

Candidemia is associated with high mortality rates in the United States. In 2016, the Infectious Diseases Society of America (IDSA) provided recommendations for the management of candidemia in hospitalized patients. However, there may be further benefit with the addition of an Infectious Diseases consultation (IDC) in patients with candidemia, as seen in patients with *Staphylococcus aureus* bacteremia.

**Methods:**

We conducted a retrospective cohort study to assess the impact of IDC on adult hospitalized patients with ≥ 1 blood culture positive for *Candida* species admitted from January 1, 2016 to December 31, 2021. Patients who were deceased or discharged prior to culture positivity, deceased within 24 hours of culture collection, or transitioned to comfort care within 48 hours from the time of positive blood cultures were excluded. The primary outcome was in-hospital and 30-day mortality. Secondary outcomes included duration of candidemia, time to active antifungal therapy, source control, and adherence to IDSA candidemia guideline recommendations.

**Results:**

A total of 195 patients met study inclusion criteria, of which 74.9% had an IDC. In-hospital and 30-day mortality were numerically higher in the non-IDC group, but were not statistically significant (24.7% vs. 34.7%, p=0.17; 18.5% vs 30.6% p=0.07). Duration of candidemia was similar between groups, but trended longer in the non-IDC group (3.25 vs. 3.90 days, p=0.28). Median time to source control, when achieved, was four days in both groups with a higher rate of obtaining source control seen in the IDC group (56.2% vs. 49%, p=0.38). Overall compliance to IDSA guidelines was higher in IDC group versus non-IDC group (47.26% vs. 42.86%, p=0.59).

**Conclusion:**

In our study, IDC was associated with lower in-hospital and 30-day mortality rates in hospitalized patients with candidemia, although not statistically significant. IDC may also be associated with shorter duration of candidemia, higher rate of achieving source control, and higher rates of adherence to the IDSA candidemia guideline recommendations, including using echinocandins as empiric therapy, repeating blood cultures, and conducting eye examinations. A larger study is warranted to solidify the findings of this study.

**Disclosures:**

**All Authors**: No reported disclosures

